# The impact of the COVID-19 pandemic on final year medical students in the United Kingdom: a national survey

**DOI:** 10.1186/s12909-020-02117-1

**Published:** 2020-06-29

**Authors:** Byung Choi, Lavandan Jegatheeswaran, Amal Minocha, Michel Alhilani, Maria Nakhoul, Ernest Mutengesa

**Affiliations:** 1 The Hillingdon Hospitals NHS Foundation Trust, Uxbridge, UK; 2 Department of Medicine, Hillingdon Hospital, Pield Heath Rd, Uxbridge, UB8 3NN UK; 3 Dana Farber Cancer Institute, Boston, USA; 4 Department of Informatics and Analytics, Dana-Farber Cancer Institute, 450 Brookline Ave, Boston, MA 02215 USA

**Keywords:** COVID-19, Medical education, Students, Assistantship

## Abstract

**Background:**

The coronavirus disease (COVID-19) global pandemic has resulted in unprecedented public health measures. This has impacted the UK education sector with many universities halting campus-based teaching and examinations. The aim of this study is to identify the impact of COVID-19 on final year medical students’ examinations and placements in the United Kingdom (UK) and how it might impact their confidence and preparedness going into their first year of foundation training.

**Methods:**

A 10-item online survey was distributed to final year medical students across 33 UK medical schools. The survey was designed by combining dichotomous, multiple choice and likert response scale questions. Participants were asked about the effect that the COVID-19 global pandemic had on final year medical written exams, electives, assistantships and objective structured clinical examinations (OSCEs). The survey also explored the student’s confidence and preparedness going into their first year of training under these new unprecedented circumstances.

**Results:**

Four hundred forty students from 32 UK medical schools responded. 38.4% (*n* = 169) of respondents had their final OSCEs cancelled while 43.0% (*n* = 189) had already completed their final OSCEs before restrictions. 43.0% (*n* = 189) of assistantship placements were postponed while 77.3% (*n* = 340) had electives cancelled. The impact of COVID-19 on OSCEs, written examinations and student assistantships significantly affected students’ preparedness (respectively *p* = 0.025, 0.008, 0.0005). In contrast, when measuring confidence, only changes to student assistantships had a significant effect (*p* = 0.0005). The majority of students feel that measures taken during this pandemic to amend their curricula was necessary. Respondents also agree that assisting in hospitals during the outbreak would be a valuable learning opportunity.

**Conclusions:**

The impact on medical student education has been significant, particularly affecting the transition from student to doctor. This study showed the disruptions to student assistantships had the biggest effect on students’ confidence and preparedness. For those willing to assist in hospitals to join the front-line workforce, it is crucial to maintain their wellbeing with safeguards such as proper inductions, support and supervision.

## Background

Coronavirus disease 2019 (COVID-19) is a potentially severe acute respiratory infection caused by the novel severe acute respiratory syndrome coronavirus 2 (SARS-CoV-2). It has been declared a pandemic by the World Health Organization (WHO) [1].

SARS-CoV-2 is highly transmissible, currently estimated to be 2 times more so than seasonal influenza [2, 3]. This greater transmissibility has resulted in an unprecedented public health response from the United Kingdom (UK) government, who have enforced social distancing at both the individual and population level. Measures introduced include nationwide school closures, banning of public events, self-isolation for symptomatic individuals, and most recently ‘lockdown’: legislation restricting non-essential public gatherings such as public events; the closure of businesses, educational and public institutions; and stay-at-home orders aside from essential tasks and exercise. This governmental response has been guided by predictive modelling which has shown that these measures may slow the spread of COVID-19 to the most vulnerable populations and ensure a manageable caseload in the National Health Service (NHS) [4].

The introduction of these measures in the UK has had a profound impact on the economy, NHS, and in particular the education sector. Universities have halted non-essential services, with many restricting campus-based teaching, and continuing courses through online resources. Some are making use of remote online assessment [5]. Final year medical students are a group who have been uniquely affected by these changes. UK medical schools are responsible to ensure their graduates meet the outcomes set out by the General Medical Council (GMC) to practise safely as a doctor. In 2009, the General Medical Council UK (GMC) published guidance on outcomes medical students are expected to meet entitled ‘Tomorrow’s Doctors’ [6], which was updated in 2018 as ‘Outcomes for Graduates’ [7]. As a prerequisite to meet these outcomes, final year medical students are required to pass end of year examinations, complete a student assistantship and often undertake a medical elective.

### Definitions of terminology

The UK Foundation Programme (UKFP)

- This is the UK postgraduate training programme for new medical graduates. It is a 2-year programme consisting of Foundation Year 1 (FY1) which is equivalent to an internship, and Foundation Year 2 (FY2) which is an additional year of experience before starting specialty training [8].

Student assistantship

- This is defined as a placement during medical school, where final year medical students, under supervision, undertake most of the duties and responsibilities of an FY1 doctor [6].

Elective

- These are placements where medical students can learn by assisting in real healthcare situations and can complete an elective in the UK or abroad. These can be organised by the students themselves to explore areas of interest and often takes place during the penultimate or final year of the degree [9].

Many of these assessments and placements have been affected as a result of the COVID-19 pandemic, with some being cancelled, postponed, or adjusted in format. There have been calls for final year medical students to either volunteer or have their General Medical Council (GMC) provisional registration fast-tracked, so that they can assist as part of the workforce [10]. The aim of our study is to identify the impact of the COVID-19 outbreak on final year medical students’ examinations, electives and assistantship placements and the subsequent effect on preparedness and confidence of students going into FY1. This will provide valuable insight for medical schools on how the pandemic has affected medical education and recommendations moving forward.

## Methods

An online survey was developed and distributed to 33 UK medical schools with an estimated cohort of 7399 final year medical students [11]. A 10-item questionnaire (Additional file 1) was designed, combining dichotomous, multiple choice and Likert response scale questions with the option for respondents to provide further free text responses for each question.

Prior to sending out the survey, a focus group was conducted for the study at our local hospital with clinical teaching fellows and current FY1 doctors, to elucidate aspects of the transition period from student to doctor that could potentially be impacted by the COVID-19 pandemic. This process facilitated the survey design to ensure questions were structured, clear and cohesive.

In the survey, the final year medical students were asked to self-assess on a 5-point Likert scale of agreeableness against specific questions [12]. Google Forms was the online platform chosen to deliver the self-administered surveys. Google Forms requires participants to be signed-in to a Google account to complete the survey which prevented multiple entries from individual respondents. In order to maintain anonymity, the email addresses used were not collected. On 22nd March, an invitation to participate in the survey was sent out to final year medical students across the UK alongside an introductory message. The survey was distributed using social media groups such as foundation school groups. At the time of the survey being distributed, clear instruction was given to only participate if individuals were graduating in the academic year of 2019–2020 and also starting FY1 in August 2020. Study participation was voluntary and no identifying information was collected.

The responses to the questionnaire were collated with two key outcomes being the students’ preparedness for FY1 and their confidence if asked to assist in hospital earlier than expected. Preparedness and confidence were assessed statistically as subjective variables against:

(i) whether Objective Structured Clinical Examinations (OSCEs) took place or not,
(ii) whether written exams took place or not,
(iii) whether students were able to do their final year assistantships and
(iv) whether students’ electives were cancelled or not.

Data obtained from the survey was subject to analysis using R version 3.6.1. Fisher’s exact test was used to assess non-random differences between the medical school cohorts for the variables of interest. Funnel plots demonstrate medical school effect size and describe the average percentage of the responses for not being prepared for FY1 and the average percentage of participants that felt less confident to assist in hospitals early. 95 and 99% confidence interval limits were used to assess the heterogeneity of the respondents’ effects from medical schools.

The NHS Research Ethics Committee tool provided by the Medical Research Council determined that ethical approval was not required for this study [13].

## Results

**Fig. 1:**
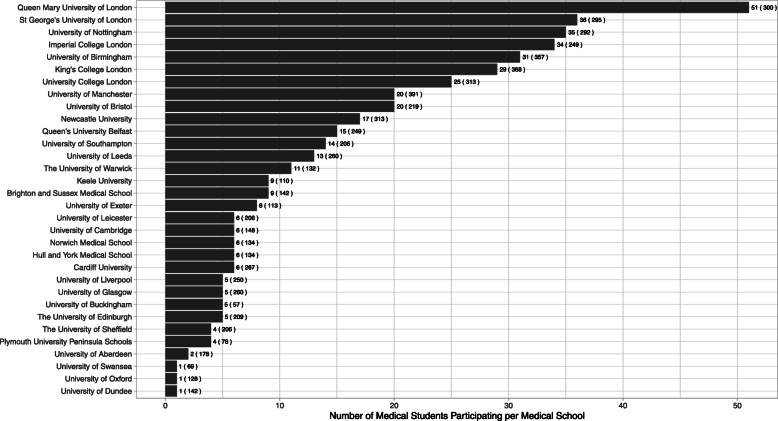
List of respondents per medical schools. Figure shows the list of the 32 UK medical schools with final year medical students participating in our survey. The number of respondents (*n* = 440) per medical school is superimposed over the number of graduates per medical school based on 2019 data from the UK foundation programme

In this study, a total of 440 students, on average 13.75 ± 12.7 participants per medical school, from 32 out of 33 UK medical schools responded to the survey. Figure 1 shows the number of responses per medical school. The number of final year medical students graduating per year is estimated to be just over 8000 [11]; data for the 2020 cohort was not available at the time of publishing. Based on the data from 2019 [11], where 7399 final year medical students in the UK were allocated to the foundation programme, our estimated response rate was 5.9%.

**Fig. 2:**
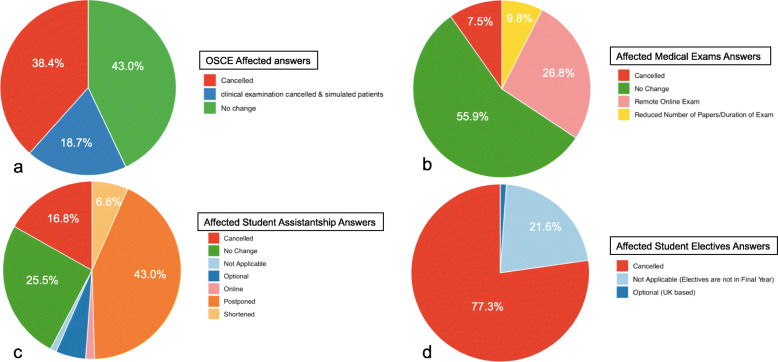
Pie-charts showing the impact of COVID-19 on medical school OSCEs (**a**), written exams (**b**), assistantships (**c**) and electives (**d**). For (**a**-**d**), *n* = 440

The impact of COVID-19 on final year examinations, electives and student assistantships is shown in Fig. 2. With regard to OSCEs, 43.0% (*n* = 189) reported no change due to examinations taking place prior to the lockdown. 38.4% (*n* = 169) had their examinations cancelled, whilst the remaining 18.6% (*n* = 82) had either simulated patients or the OSCE stations requiring patient contact cancelled (Fig. 2a). For written examinations, 55.9% (*n* = 246) reported no change whilst 9.8% (*n* = 43) reported cancellations. A small proportion of students had either the duration of their written examination or the total number of written papers reduced in size (7.5%; *n* = 33). For the remainder of the students, (26.8%; *n* = 118), the written examinations were completed online remotely (Fig. 2b).

A large number of medical elective placements were predictably cancelled (77.3%; *n* = 340) due to travel restrictions. 21.6% (*n* = 95) of students had fortunately completed their elective periods whilst 1.1% (*n* = 5) reported that they had been offered an optional UK based elective (Fig. 2c). The student assistantship period was also heavily disrupted with only a quarter of respondents reporting no change (25.5%; *n* = 112). 16.8% (*n* = 74) reported cancellations, 6.6% (*n* = 29) reported placements were shortened, 43.0% (*n* = 189) had their assistantship postponed. Surprisingly, 5.5% (*n* = 24) reported that their assistantship was optional and 1.6% (n = 7) had it replaced with formative online modules. Whether postponed assistantships will go ahead is still unknown, as it is not yet clear when the lockdown period will end.

**Fig. 3:**
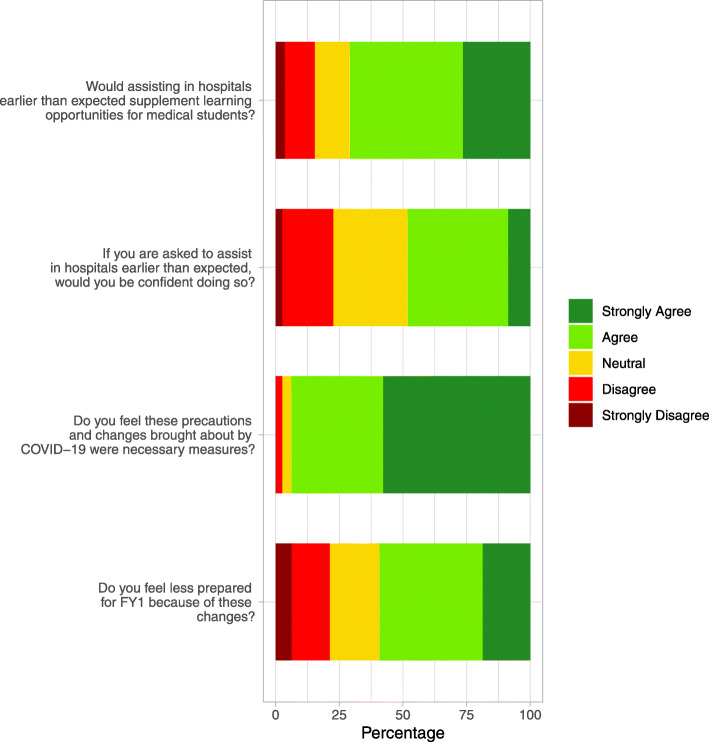
Stacked bar chart of Likert questions. The figure shows 4 bar charts describing how final year medical students answered the Likert scale questions in the survey. The 4 questions assessed in descending order: whether assisting in hospitals earlier than expected supplemented final year medical students’ learning; whether students would be confident in assisting in hospitals earlier than expected; whether students felt that the precautions taken because of COVID-19 were necessary; and whether students felt less prepared because of these changes

Within this cohort of final year medical students, 49.5% (*n* = 218) had been asked to assist or start working in hospitals, before the anticipated schedule, to help the NHS workforce during this pandemic. The respondents were further assessed using Likert scales of agreeables across 5 items (Fig. 3). 57.8% (*n* = 254) ‘Strongly Agree’ and 36.1% (*n* = 159) ‘Agree’ that measures taken by medical schools to amend their curricula were necessary. With respect to confidence, 8.7% (*n* = 38) ‘Strongly Agree’ and 39.5% (*n* = 174) ‘Agree’ that they would feel confident in starting prior in hospitals than predicted. When asked whether assisting in hospitals during the COVID-19 outbreak would supplement their learning opportunities, the majority of respondents ‘Strongly Agree’ (26.5%; *n* = 116) or ‘Agree’ (44.5%; *n* = 196). When asked if respondents felt less prepared for FY1, due to the disturbance caused by COVID-19, 18.6% (*n* = 82) ‘Strongly Agree’, 40.7% (*n* = 179) ‘Agree’ and 19.3% (*n* = 85) remained ‘Neutral’.

**Fig. 4:**
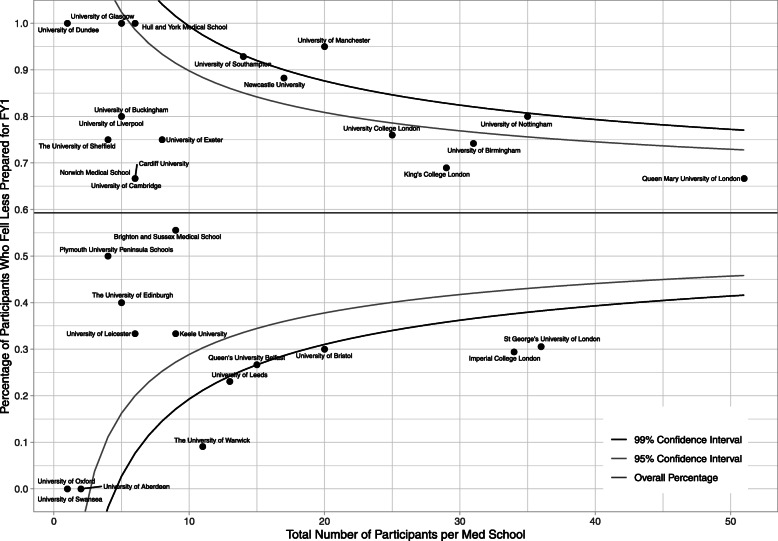
Funnel Plots Showing Effect Sizes and Heterogeneity on a Medical School Level: Students Feeling Less Prepared for FY1. Funnel plot showing the percentage of final year medical students from different medical schools who felt less prepared to start FY1. University of Manchester, St George’s University of London, Imperial College London, University of Bristol, University of Leeds and The University of Warwick medical schools have results outside the 99.9% confidence limits of the overall mean and this may be regarded and significantly different from the global results

**Fig. 5:**
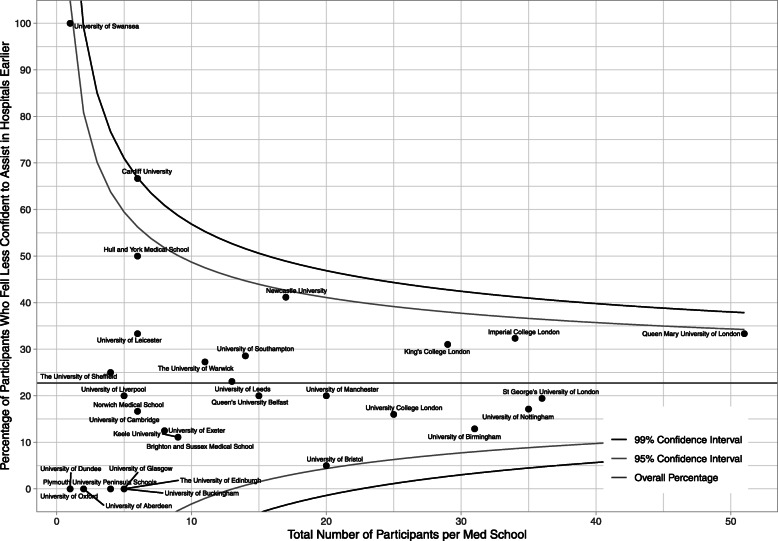
Funnel Plots Showing Effect Sizes and Heterogeneity on a Medical School Level: Students Feeling Confident to Assist in Hospitals Earlier. Funnel Plot showing the percentage of final year medical students from different medical schools who felt less confident to assist in hospitals earlier

Funnel plots were used to assess students’ confidence in starting earlier than expected as well as preparedness going into FY1. Students that answered ‘Agree’ or ‘Strongly Agree’ to be prepared for the transition into FY1 were used for Fig. 4, and on average, 59.32% of these students felt unprepared for FY1. Furthermore, respondents from 11 medical schools were outside of the 95% confidence interval limits of the overall mean for preparedness in starting as an FY1. Of these 11, 6 were outside of the 99% confidence interval limit (Fig. 4). In contrast, respondents of only one medical school were found to be outside of the 95% confidence interval limits of the overall mean when measuring confidence in starting as an FY1 (Fig. 5). Overall, there was an average of 22.7% of respondents who did not feel confident starting in hospitals earlier than expected. Skewness in the curve is affected by medical schools that had fewer respondents with respect to other schools, such as University of Swansea, which only had 1 participant in the study.

The consequent levels of confidence, as well as preparedness, due to the impact of OSCEs, written examinations, electives and assistantships were analysed. Fisher’s exact test was used to assess differences in the cohorts for our variables of interest. When considering students’ preparedness for FY1, the changes to OSCE examinations, student assistantships, and written examinations had a significant effect (*p* = 0.025, *p* = 0.0005 and *p* = 0.008 respectively). Student electives however, did not have a significant effect on preparedness for FY1 (*p* = 0.427) (Table 1). When evaluating confidence in starting in hospital earlier than expected, the only statistically significant impact was the disruption of student assistantships (*p* = 0.0005) (Table 2).

**Table 1:**
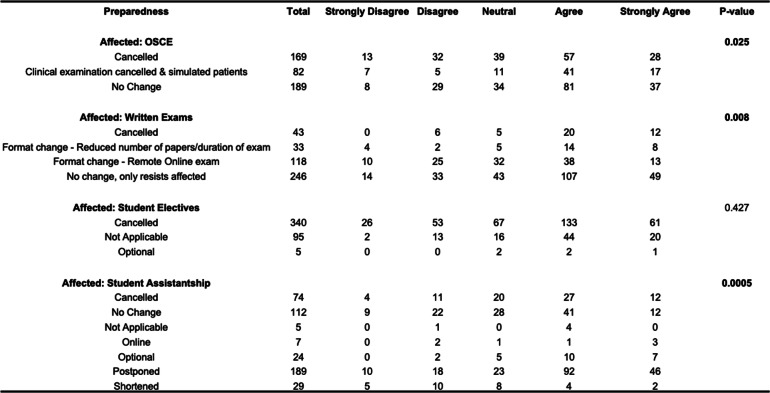
Factors affecting Preparedness for FY1

Table showing how different factors (OSCE, written exams, students electives and assistantships) affected students’ preparedness to start FY1. Fisher’s exact test was used to produce a *p*-value. The analysis shows that final year OSCEs, written exams and students assistantships significantly affected final year medical students’ preparedness to start FY1 (respectively: *p* = 0.025, 0.008, 0.0005)

**Table 2:**
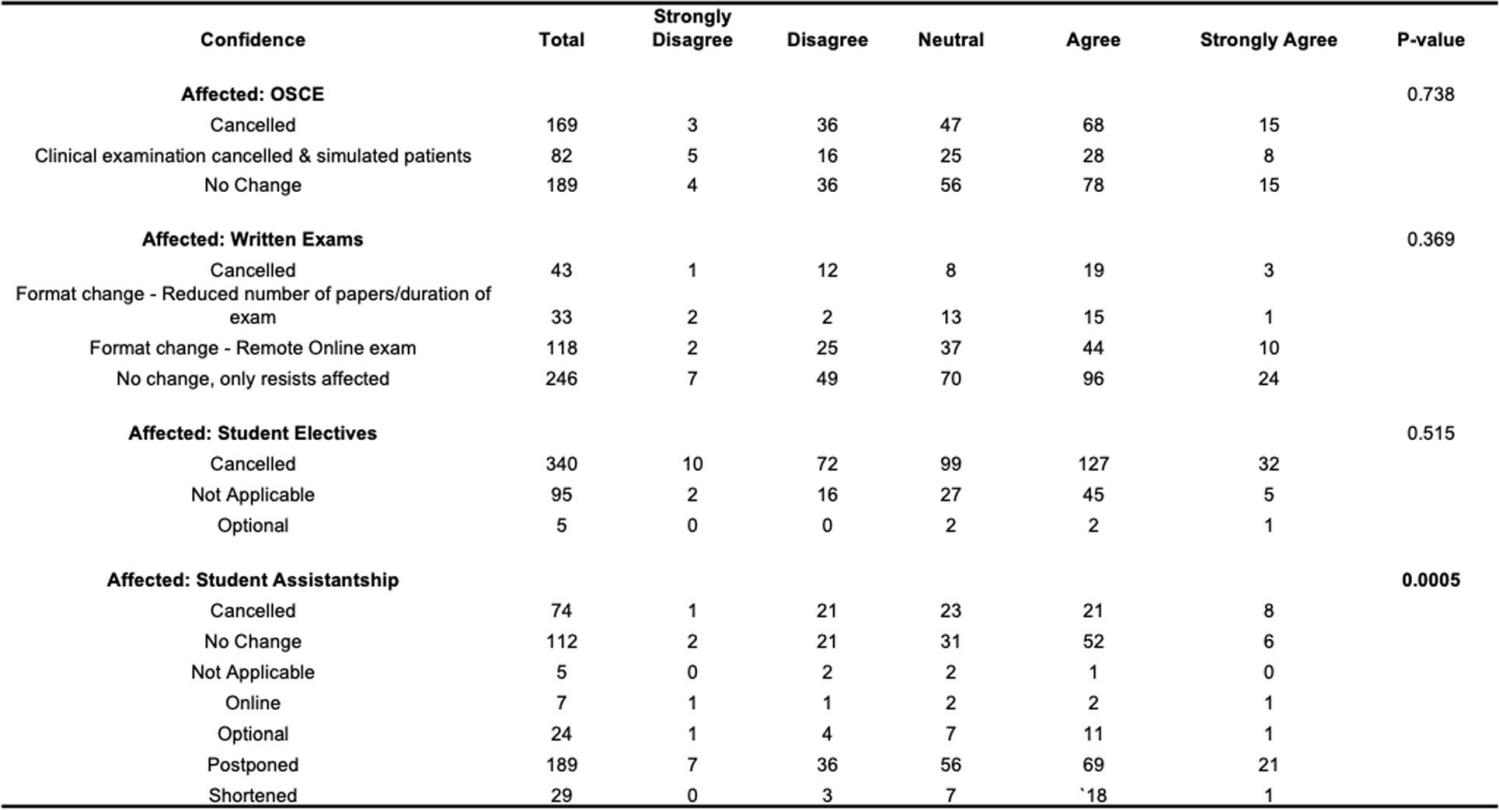
Factors affecting the Confidence to Assist in Hospitals Earlier

Table showing how different factors (OSCE, written exams, students electives and assistantships) affected students’ confidence to assist in hospitals earlier than expected. Fisher’s exact test was used to produce a p-value. The analysis shows that only final year students’ assistantship significantly affected final year medical students’ confidence to start assisting in hospital work earlier than expected (*p* = 0.0005)

## Discussion

The aim of our study is to identify the impact of the COVID-19 outbreak on final year medical students’ examinations, electives and assistantship placements and the subsequent effect on preparedness and confidence of students going into FY1. Current student attitudes toward the pandemic were also explored. The results of the survey show that respondents had a utilitarian opinion on the impact of COVID-19; almost all respondents (93.9%) felt changes that had been made were necessary measures during this pandemic. 77.3% of respondents had electives cancelled; although disappointing to many, students recognised that worldwide travel restrictions were necessary. Statistical analysis highlights key issues related to training that should be addressed in the immediate term. Furthermore, better ways to support students to transition into the workforce in the long-term is an essential element that needs consideration.

### Transition from student to doctor

Amongst respondents across UK medical schools, 16.8% reported that student assistantship had been formally cancelled, while 43.0% reported that assistantships were postponed. Fortunately, 25.5% had no change to their assistantship as they were completed prior to the introduction of COVID-19-related nationwide restrictions. Figures 4 and 5 demonstrate that there are respondents from medical schools who feel less prepared or less confident in starting in hospitals earlier than expected. There are, on average, 59.3% of students who felt less prepared whilst 22.7% felt less confident. For confidence, the asymmetric trend of Fig. 5 shows that the majority of respondents across UK medical schools felt confident starting in hospitals earlier than expected, with respondents from only 1 medical school being outside the 95% confidence interval. In contrast, when considering preparedness, there were a greater number of respondents per medical school who felt less prepared. Further to that, a greater number deviated from the average, with respondents from 11 medical schools being outside of the 95% confidence interval and 6 outside of the 99% confidence interval. This indicates a significant deviation from the overall cohort in this survey, and overall, more individuals across UK medical schools felt less prepared in assisting in hospitals. This highlights the importance of the transition period for incoming FY1 doctors.

The aim of the student assistantship is to provide students with an enhanced opportunity to aid the transition from student to doctor and to familiarise with common tasks they will eventually take on as an FY1 doctor [8]. In contrast to traditional clinical rotations, assistantships are integrated in such a way that empowers students with greater integration within the team, to help develop clinical, practical and administrative skills and ownership of responsibility in a professional capacity [14]. Analysis showed that the interruption to student assistantship significantly impacted both on preparedness for FY1 and confidence in starting sooner than expected for final year medical students (*p* = 0.0005 and *p* = 0.0005 respectively). In contrast, the disturbance to electives was not significant for either preparedness, or confidence (*p* = 0.427 and *p* = 0.515 respectively). This suggests that for final year medical students, assistantship plays a key role, more so than the elective period, in the transition from student to doctor. Given that 70.9% of students either ‘Agree’ or ‘Strongly Agree’ that assisting in hospitals prior to formally starting work as a doctor would supplement learning opportunities lost due to COVID-19, it is clear that the student body views this transition period as an important part of their professional development.

This pandemic has created the opportunity for early provisional registration to practice as doctors for students. Although likely to be taken up by a number of final year medical students eager to support the frontline workforce, it should be recognised that there is a tension between safeguarding education and responding to the demands on the health service [15].

Whilst it is encouraging to see a cohort so willing and adaptable, it is important to take into consideration that the students have lost out on months of intensive preparation in transitioning from student to doctor. The UK Medical Schools Council has advised that the first responsibility of medical students is to continue their education and to “not jeopardise their readiness to qualify in the future by taking on too many additional responsibilities” [16]. Given the unprecedented interruptions caused by the pandemic, it is clear that students should not be brought in to enhance the workforce without proper inductions, clear guidance on working within their competencies, pastoral support, and appropriate remuneration for their time. This is essential to maintaining both patient care and student wellbeing.

For medical schools deploying students to assist in hospitals during the COVID-19 outbreak, this is an opportunity to evaluate the involvement, responsibilities and roles that they are given. Medical students can be given targeted volunteering opportunities that supplement their educational needs. For clinical medical students volunteering on hospital wards, this is an opportunity to focus on becoming integrated into the team and learning through mentorship by shadowing doctors completing their clinical activities [17]. Throughout this, students will not be ‘working’ with the added pressure of ensuring specific learning objectives are met and without the need for being signed off for various competencies [18, 19]. By becoming integrated into the team, with prolonged clinical supervision and feedback, medical schools can assess whether these volunteering opportunities mirror that of alternative educational models such as the Longitudinal Integrated Clerkship (LIC). LIC as an educational model has grown in popularity worldwide, being most widely utilised in primary care [20]. The LIC model differs from student assistantship as it is a long-term placement. This provides students with prolonged involvement and continuity in the care of patients which promotes ownership of care and responsibility whilst having adequate supervision [21, 22]. Further benefits of LIC are development of student empathy and patient-centredness due to their continued involvement throughout a patient’s care. Dundee School of Medicine was the first to introduce a comprehensive LIC lasting for a whole academic year [20]. Other institutions such as Imperial College and Hull York Medical School have piloted LIC programmes that run in primary and secondary care. It remains to be seen within the UK whether LIC will be introduced more widely into medical school curricula, but early student feedback is positive [23, 24]. The COVID-19 pandemic may provide valuable insight into assessing how clinical placements can be improved within the medical curriculum in the UK.

### Online assessments and education

A significant proportion of final year students had already taken written and clinical examinations prior to the COVID-19 outbreak, or indeed prior to implementation of social distancing, and subsequent university closures. For OSCEs, just under half of UK medical schools had already completed them, and around a third had these clinical examinations cancelled. Four medical schools adjusted them by using actors rather than real patients.

Similarly, written examinations were completed prior to the interruptions in more than half of UK medical schools. Analysing the effect of exam disruptions on preparedness for both OSCEs and written examinations showed a statistically significant impact on preparedness (*p* = 0.0005). In contrast the exam disturbances were not significant when it came to confidence (written exams *p* = 0.369, OSCEs *p* = 0.738). This may suggest that students perceive clinical knowledge developed through examination as an important contributing factor in their preparedness to work, whilst their clinical confidence is developed in ways other than through academic assessment.

Interestingly, in a first for UK medical schools, 6 medical schools changed the written final year examination to be done remotely. If the COVID-19 lockdown continues, it may be possible that re-sitting of examinations may also be online. At Imperial College London, the online assessment consists of an open book examination of 150 questions, with 72 s to answer each one. Questions were randomised to prevent helping each other. Students were presented with simulated patients and through provided history, examination and investigation findings were required to work through questions [5]. If psychometric analysis of the data from these remote examinations appear to be comparable with that of closed book examinations, it may accelerate an incoming online era of medical student assessment. Formative online assessments are already widespread in medical education; this facilitates learners and teachers to identify areas of weakness and provide prompt feedback for continued development. In contrast to formative assessments, summative assessments, such as medical school final year examinations, are high-stakes; with consequences of students passing or failing with the purpose of determining if students are ready to advance to the next stage of their training or grant graduation [25]. The online summative assessments enforced as a result of the COVID-19 pandemic at certain medical schools may provide invaluable data for the evaluation of the efficacy of online summative assessment moving forward.

Despite the closures of university campuses, medical education has not been entirely suspended due to online platforms. Virtual online communication platforms (e.g. Zoom, Microsoft Teams, Skype) have enabled lectures and small group teaching to continue, whilst formative assessments have enabled continued learning and development. Online software has provided an important solution to continue medical education despite the disruptions of COVID-19.

Online assessment and education presents its own challenges. Firstly, reliability of online assessment systems, particularly network connectivity, needs to be robust to be used confidently. Furthermore, some students may not have a home environment conducive to sitting an examination, may have difficult personal circumstances at home, or have barriers of access to adequate online facilities [5]. Moving forward, a robust system to ensure standardisation for student’s remote examination setting and clear guidance on extenuating circumstances should be made. Although online assessments and teaching methods have become more prominent in the past decade, the COVID-19 pandemic has enforced circumstances which may accelerate their widespread use in medical education.

### Limitations

Our study has some limitations which should be stated. The first limitation is the retrospective nature of the study design. Although the current perspectives of final year medical students have been established, there was no follow-up period for the respondents. Consequently, the long term impact of COVID-19 on the transition period from student to doctor cannot yet be determined.

Another limitation is the difference in the number of participants across medical schools (Fig. 1). This variation between medical schools means that our data does not represent the entire cohort of final year medical students who will be starting FY1 in 2020. Based on the 2019 UK foundation programme data, the overall response rate for our survey was estimated to be only 5.9% of the total national cohort of final year medical students going into FY1. It is recognised that this may mean the responses captured reflect a subset of students who feel either more or less confident and/or prepared, and as such the external validity of this study is limited.

In addition, the responses for adjustments to examinations for each medical school were compared across all respondents for consistency. Given that there were little to no discrepancies between the responses per medical school, a process of verification with medical schools was not undertaken. We recognise this as a limitation as there remains a potential for inaccuracies in respondents’ recollection and reporting of these examination changes.

### Recommendations

1. Fast tracked graduation of medical students should not compromise on supervision, pastoral support or appropriate induction in order to maintain the wellbeing of both newly graduating doctors and patients.
2. Alternative models of clinical education (which build on the benefits offered by assistantship) in the final year of study, such as the Longitudinal Integrated Clerkship, should be explored for more widespread implementation nationally, to help optimise the transition from student to doctor.
3. There is a need for robust guidelines on continued educational development for medical students during times of crisis and future pandemics.
  - Medical students’ health and safety must not be jeopardised to supplement the health workforce.
  - Medical students’ priority is their continued education to ensure they meet learning objectives required to graduate as doctors and meet standards set out by the GMC.
4. The use of online platforms for both education and assessment should be optimised across medical schools.
  - Online platforms provide medical schools with an avenue to maintain the provision of medical education remotely, in order to maintain the professional development of medical students as set out by the GMC.
  - For the wider introduction of remote online summative assessments as part of the medical curricula, there must be robust systems in place to ensure fairness of these assessments.
  - It is necessary as part of preparation for future pandemics or other disruptions to medical education, to develop the capacity to seamlessly carry out remote final year examinations.

## Conclusion

The impact of COVID-19 on final year medical student education has been significant in the UK. The majority of students feel less prepared for beginning work as a doctor, with this study showing that the disruptions to student assistantships had a significant impact on preparedness. Despite being unprepared, many students feel confident in joining the workforce during this pandemic. Medical schools and employers should not disregard the COVID-19-related interruptions to the final months of preparation for this cohort of students and it is essential their wellbeing is not compromised. Changes enforced by this pandemic provide a key opportunity to evaluate alternative modes of medical education and assessment, including novel online summative assessments. Many lessons will be learnt throughout the months of this pandemic and educators will be better placed to act promptly in future health crises or disruptions to medical education.

## Supplementary information

**Additional file 1.** 10 point questionnaire that was designed and sent to final year medical students. Aim of this survey was to identify changes to assessments as a consequence of COVID-19 and how this impacted their attitudes, confidence and preparedness in starting as a doctor.

## Data Availability

The datasets used and/or analysed during the current study are available from the corresponding author on reasonable request.

## Abbreviations

COVID-19: Coronavirus disease 2019
FY1: Foundation Year 1
GMC: General Medical Council
LIC: Longitudinal Integrated Clerkship
OSCE: Objective Structured Clinical Examination
WHO: World Health Organization
