# Supplementary material for: The impact of the COVID-19 pandemic on final year medical students in the United Kingdom: a national survey
Source: BMC Med Educ. 2020 Jun 29;20:206. doi: 10.1186/s12909-020-02117-1 (PMC7323883; doi:10.1186/s12909-020-02117-1)
Supplement: Supplementary file 1 — Additional file 1. 10 point questionnaire that was designed and sent to final year medical students. Aim of this survey was to identify changes to assessments as a consequence of COVID-19 and how this impacted their attitudes, confidence and preparedness in starting as a doctor. [file 12909_2020_2117_MOESM1_ESM.docx]

**Additional file 1.**

**The Impact of the COVID-19 Pandemic on Final Year Medical Students in the United Kingdom: a National Survey**

The impact of COVID-19 is currently being felt across the NHS, and is now also affecting medical education. This survey is for final year medical students studying at UK medical schools. The aims of this survey are to identify changes that have been made to final year medical curricula because of COVID-19, and the impact this is having on final year medical students.

Participation in this survey is voluntary and any information will be kept anonymous.

1. **Which UK medical school do you study at?**
   1. Brighton and Sussex Medical School
   2. Cardiff University
   3. Hull and York Medical School
   4. Imperial College London
   5. Keele University
   6. King’s College London
   7. Newcastle University
   8. Norwich Medical School
   9. Plymouth University Peninsula Schools
   10. Queen Mary University of London
   11. St George’s University of London
   12. The University of Edinburgh
   13. The University of Sheffield
   14. The University of Warwick
   15. University College London
   16. University of Aberdeen
   17. University of Birmingham
   18. University of Buckingham
   19. University of Bristol
   20. University of Cambridge
   21. University of Dundee
   22. University of Exeter
   23. University of Glasgow
   24. University of Lancaster
   25. University of Leeds
   26. University of Leicester
   27. University of Liverpool
   28. University of Manchester
   29. University of Nottingham
   30. University of Oxford
   31. University of Southampton
   32. University of Swansea
2. **How has COVID-19 affected final year OSCEs at your medical school? Tick all options that apply below:**
   1. Postponed
   2. Cancelled
   3. Format changed (if so, please elaborate under Other)
   4. No change
   5. Other
3. **How has COVID-19 affected final year written exams at your medical school? Tick all options that apply below:**
   1. Postponed
   2. Cancelled
   3. Format changed (if so, please elaborate under Other)
   4. Venue changed (if so, please elaborate under Other)
   5. No change
   6. Other
4. **How has COVID-19 affected medical student assistantship training at your medical school? Tick all options that apply below:**
   1. Postponed
   2. Cancelled
   3. Format changed (if so, please elaborate under Other)
   4. No change
   5. Other
5. **How has COVID-19 affected medical student electives at your medical school? Tick all options that apply below:**
   1. Postponed
   2. Cancelled
   3. Location changed (if so, please elaborate under Other)
   4. No change
   5. Not applicable (medical electives are not in final year)
   6. Other
6. **Do you feel less prepared for FY1 because of these changes?**
   1. Strongly Agree
   2. Agree
   3. Neutral
   4. Disagree
   5. Strongly Disagree
7. **Do you feel these precautions and changes brought about by COVID-19 were necessary measures?**
   1. Strongly Agree
   2. Agree
   3. Neutral
   4. Disagree
   5. Strongly Disagree
8. **Have you been asked to assist in hospitals earlier than expected?**
   1. Yes
   2. No
9. **If you are asked to assist in hospitals earlier than expected, would you be confident doing so?**
   1. Strongly Agree
   2. Agree
   3. Neutral
   4. Disagree
   5. Strongly Disagree
10. **Assisting in hospitals earlier than expected would supplement learning opportunities for medical students where electives, assistantships and placements have been cancelled.**
    1. Strongly Agree
    2. Agree
    3. Neutral
    4. Disagree
    5. Strongly Disagree
